# Disseminating Childhood Home Injury Risk Reduction Information in Pakistan: Results from a Community-Based Pilot Study

**DOI:** 10.3390/ijerph10031113

**Published:** 2013-03-15

**Authors:** Aruna Chandran, Uzma Rahim Khan, Nukhba Zia, Asher Feroze, Sarah Stewart de Ramirez, Cheng-Ming Huang, Junaid A. Razzak, Adnan A. Hyder

**Affiliations:** 1 International Injury Research Unit (IIRU), Department of International Health, Johns Hopkins University Bloomberg School of Public Health, Baltimore, MD 21205, USA; E-Mails: nzia@jhsph.edu (N.Z.); sderamirez@jhmi.edu (S.S.R.); chuang@jhsph.edu (C.-M.H.); ahyder@jhsph.edu (A.A.H.); 2 Department of Emergency Medicine, Aga Khan University, Karachi 74800, Pakistan; E-Mails: uzma.khan@aku.edu (U.R.K.); asher.feroze@aku.edu (A.F.); junaid.razzak@aku.edu (J.A.R.)

**Keywords:** unintentional injuries, home injuries, children, accidents, trauma, home visits, Pakistan

## Abstract

*Background*: Most childhood unintentional injuries occur in the home; however, very little home injury prevention information is tailored to developing countries. Utilizing our previously developed information dissemination tools and a hazard assessment checklist tailored to a low-income neighborhood in Pakistan, we pilot tested and compared the effectiveness of two dissemination tools. *Methods*: Two low-income neighborhoods were mapped, identifying families with a child aged between 12 and 59 months. In June and July 2010, all enrolled households underwent a home hazard assessment at the same time hazard reduction education was being given using an in-home tutorial or a pamphlet. A follow up assessment was conducted 4–5 months later. *Results*: 503 households were enrolled; 256 received a tutorial and 247 a pamphlet. The two groups differed significantly (*p* < 0.01) in level of maternal education and relationship of the child to the primary caregiver. However, when controlling for these variables, those receiving an in-home tutorial had a higher odds of hazard reduction than the pamphlet group for uncovered vats of water (OR 2.14, 95% CI: 1.28, 3.58), an open fire within reach of the child (OR 3.55, 95% CI: 1.80, 7.00), and inappropriately labeled cooking fuel containers (OR 1.86, 95% CI: 1.07, 3.25). *Conclusions*: This pilot project demonstrates the potential utility of using home-visit tutorials to decrease home hazards in a low-income neighborhood in Pakistan. A longer-term randomized study is needed to assess actual effectiveness of the use of allied health workers for home-based injury education and whether this results in decreased home injuries.

## 1. Background

Unintentional injuries are major causes of mortality and morbidity among children, resulting in over 875,000 deaths annually in children <18 years of age annually around the World [1]. For young children between 1 and 5 years of age, not only are unintentional injuries the leading cause of death, but they are also responsible for millions of non-fatal injuries, often leaving children with lifelong disabilities [2]. Because of the long periods of time that children spend in the home, in combination with the variety of potential hazards that are present in the household environment, a substantial proportion of childhood unintentional injuries occur in this setting [3,4,5]. As has been shown in several studies mostly focused in high-income countries (HIC), a substantial number of childhood unintentional injuries can be prevented through modifications to the home environment [6,7,8].

A variety of methods for promoting home injury prevention to caretakers of young children have been proven effective in HICs. For example, a number of recent studies offer strong evidence that home visits by community health workers or similar allied health professionals can reduce childhood injuries in HICs [9,10,11,12,13,14,15]. In addition, healthcare practitioners in most HICs have an abundance of pre-designed pamphlets and information sheets regarding methods for “safety-proofing” a home for young children [16,17]. However, few such resources for improving home safety have been tailored to a low and middle income country (LMIC) setting, and there is limited information regarding the best methods and tools for dissemination of home safety information in LMICs. This lack of research regarding the most effective method for dissemination of home injury risk and potential prevention information in LMICs has resulted in limited ways for health professionals to share knowledge with parents [2].

Pakistan is a low-income country with a population of over 176 million [18]. Studies in Pakistan have shown that the unintentional injury burden is substantial in young children, and that the majority of those occur in the home [3,19,20,21,22]. A recent randomized trial in which injury reduction counseling was provided in a home visit following a child being discharged from an emergency department showed a significant reduction in the presence of both fall and choking hazards [23]. To our knowledge, no similar studies have been done in this setting focusing on unintentional injury hazards beyond choking and falls.

This study was motivated by the need for additional evidence-based approaches for the dissemination of home injury prevention information in order to reduce childhood injury risk in LMIC. The study design and initial assessment of the prevalence of existing household risk for childhood injury in the chosen neighborhoods have been previously described [24]. This study describes the results of a pilot study comparing the difference in prevalence of home injury risks before and after the dissemination of two different hazard reduction tools: an educational pamphlet and an in-home tutorial.

## 2. Methods

### 2.1. Tool Development

Details of the development of the home hazard assessment checklist as well as two different tools containing information on identifying and correcting common home hazards that can result in unintentional injuries in young children (an educational pamphlet and an in-home tutorial) have been previously described [24]. Briefly, the home hazard assessment checklist included a detailed assessment of the presence of home injury hazards divided by room or living area as viewed by a trained data collector. Basic information highlighting common home injury hazards that were specific and relevant to this population, as well as low-cost easily implemented methods for reducing or eliminating those hazards was drafted; both the educational pamphlet and the in-home tutorial contained the same information. The educational pamphlet was designed such that the information was presented in a format not requiring the presence of a health practitioner for understanding or use. In contrast, the in-home tutorial was an interactive tool that allowed a trained data collector to provide home injury risk information and prevention ideas while walking through the house with the child’s caregiver. All tools were translated into Urdu, the local language commonly spoken in Karachi.

### 2.2. Study Design

Two neighborhoods were identified within a low-income government housing community in Karachi, Pakistan. This community was selected on the basis of a lower middle-income status of residents, homes with permanent structures, relatively high literacy level of residents, and ease of access from the local research institution. The neighborhoods were approximately ¼ mile apart, separated by several large and busy streets; previous observations by AKU staff suggest very little mixing between the two populations. One neighborhood was arbitrarily chosen as the “educational pamphlet group” and the other as the “in-home tutorial group”. The two neighborhoods were mapped, and then enrollment screening took place. The sample size for projected enrollment was calculated based on hazard prevalence shown by previous studies in Pakistan [19,23], an anticipated 10% difference in the in-home tutorial *vs.* educational pamphlet groups following the intervention, 90% power, 5% significance, 15% consent decline, and 10% loss to follow up.

Eligibility criteria for enrollment into the study included: presence of at least one child between the ages of 12 and 59 months, a caregiver who was able to read in Urdu, and a current plan to live in the same house for at least another three months. Eligibility was confirmed at the beginning of the interaction. Following confirmation of eligibility and obtaining of written informed consent, households were enrolled. If a household had more than one child between 12–59 months of age, the caregiver was asked to select one as the index child.

Three data collectors (DCs) were trained in the use of the home hazard assessment checklist as well as how to share the information contained in the educational pamphlet and in-home tutorial. For the educational pamphlet households, the DCs conducted the home hazard assessment, presented the pamphlet, and encouraged the caregiver to read it and share it with others involved in the care of the child. For the in-home tutorial households, the DCs conducted the home hazard assessment and then utilized the tutorial guide with the respondent by going room to room pointing out potential hazards and sharing ideas on how they might be corrected.

A follow up assessment using the identical tool as the initial hazard assessment was done 4–5 months following the intervention was done by the same DCs. At that time, the in-home tutorial households were also given an educational pamphlet to keep with them for future reference; the educational pamphlet households were not given an in-home tutorial at that time.

The interventions were conducted in June-July 2010, and the follow up assessments were done in November 2010. Approval for the study was obtained from the Ethical Review Committee of the Aga Khan University (Pakistan) and the Institutional Review Board of Johns Hopkins Bloomberg School of Public Health (USA).

### 2.3. Statistical Analysis

All data was entered into an EpiData database. Data analysis was done using Stata Version 10 (Statacorps LP, College Station, TX, USA). Demographic variables as well as the presence of hazards were compared between groups using the Student’s *t*-Test for continuous variables and the Fisher’s Exact Chi-Squared for dichotomous variables. Logistic regression analysis utilizing data from those households for which both pre and post information was available was used to assess the odds of change in hazard presence while controlling for several demographic variables including those that were statistically significantly different between the two groups.

## 3. Results

**Figure 1:**
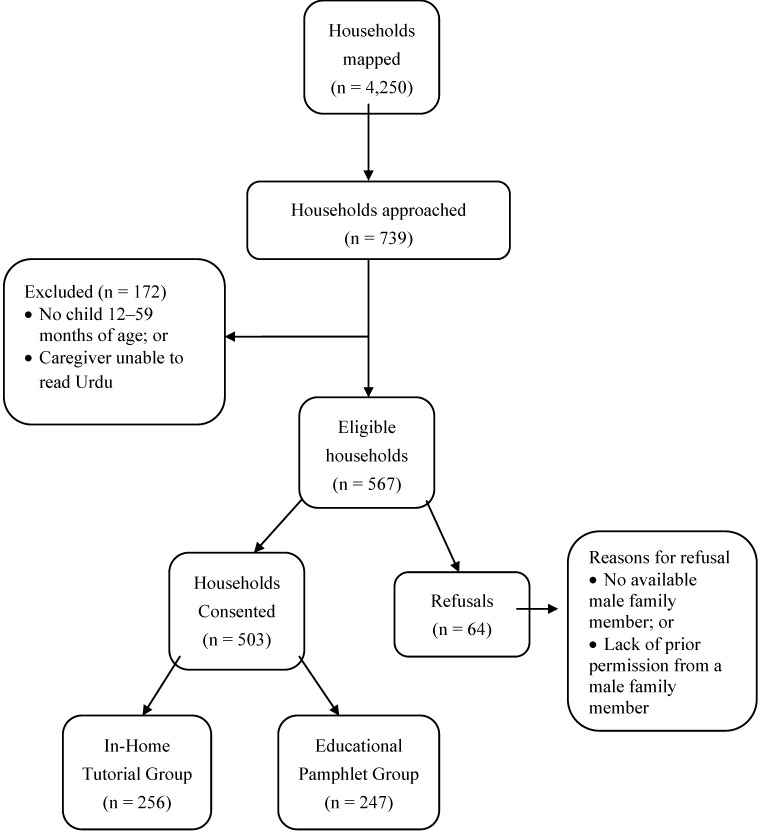
Flow chart of study household enrollment.

A total of 503 households were enrolled in the study; 256 were assigned to receive an in-home tutorial while 247 received an educational pamphlet (Figure 1). The demographic profiles of the two groups are shown in detail in Table 1. Overall, the mean age of the index children was 34.8 months (range 12 to 59 months), with a relatively equal number of males (50.7%) and females (49.3%). The respondents were nearly all female (98.8%) and occupied as housewives (93.0%). Roughly half of the respondents (53.3%) were in the 20–29 year age group. Importantly and unexpectedly, the two groups differed significantly in two demographic characteristics; the educational pamphlet group had higher levels of maternal education (*p* < 0.01) and a higher proportion of index children having the mother as the primary caregiver (*p* < 0.01). The basic injury history and risk factor characteristics of this cohort have been reported elsewhere (submitted for publication by the authors).

Table 2 outlines a comparison of the types of injury hazards present both before and after the interventions for both groups. Notably, there were significant differences between the two groups in the prevalence of several pre-intervention hazards; this is likely influenced by the demographic differences between the two groups. In both the in-home tutorial group as well as the educational pamphlet group, there was a statistically significant (*p* < 0.01) decrease in the following injury hazards after the intervention: falls (walker present), drowning (open buckets and vats/pools of water), burns (water heater, matches and stove within reach of the child), poisonings (cleaning supplies and shampoos accessible to the child), and lacerations (knives and breakable objects within reach of the child). Additionally, the in-home tutorial group had a decrease in the prevalence of medicines within reach of the child (*p* < 0.01) and open fire within reach of the child (*p* = 0.03). For reasons that are not clear, a few hazards increased in prevalence in the educational pamphlet group after the intervention as compared with before; there were a higher proportion of irons within reach of the child (*p* = 0.04) and open fires within reach of the child (*p* = 0.04).

**Table 1:** Demographic characteristics of enrollees by group.

Characteristics	In-Home Tutorial Group;	Educational Pamphlet Group;	Comparison
	N = 256 (%)	N = 247 (%)	(*p* value)
Respondent Characteristics			
*Gender*			
Female	254 (99.2)	243 (98.4)	*p* = 0.39
Male	2 (0.8)	4 (1.6)	
*Age Group*			
<20 years	6 (2.3)	8 (3.2)	*p* = 0.12
20–29 years	124 (48.4)	144 (58.3)	
30–39 years	95 (37.1)	78 (31.6)	
40–49 years	19 (7.4)	11 (4.5)	
50+ years	12 (4.7)	6 (2.4)	
*Education Level*			
<Grade 9	104 (40.6)	59 (23.9)	*p* < 0.01
≥Grade 9	152 (59.4)	188 (76.1)	
*Occupation of Mother*			
Housewife	235 (91.8)	233 (94.3)	*p* = 0.53
Other	21 (8.2)	14 (5.7)	
**Child Characteristics**			
*Mean Age (months)*	34.3	35.2	*p* = 0.42
*Gender*			
Male	131 (51.2)	124 (50.2)	*p* = 0.83
Female	125 (48.8)	123 (49.8)	
*Child’s Primary Caregiver*			
Mother	218 (85.2)	233 (94.3)	*p* < 0.01
Father	9 (3.5)	2 (0.8)	
Other	29 (11.3)	12 (4.9)	

When directly comparing the two interventions, the homes that received an educational pamphlet had a lower prevalence of several hazards than those homes that received an in-home tutorial, including the presence of open buckets of water (*p* = 0.02), a stove within reach of a child (*p* < 0.01), matches within reach of a child (*p* < 0.01), inappropriate labeled cooking fluid containers (*p* < 0.01), cleaning supplies within reach of a child (*p* < 0.01), and knives within reach of a child (*p* < 0.01) (Table 2). Conversely, the in-home tutorial group had a slightly lower prevalence of accessible rooftops without railings, iron within reach of the child, medicines within reach of a child, and breakable objects accessible to the child; however, these differences were not statistically significant. Of note, many of these differences were present between the two groups prior to the implementation of the intervention.

**Table 2:** Comparison of presence of hazard pre and post intervention by group.

	In Home-Tutorial Group			Educational Pamphlet Group		
	Pre-Intervention	Post-Intervention	Comparison	Pre-Intervention	Post Intervention	Comparison
	N = 256 (%)	N = 215 (%)	(*p* value)	N = 247 (%)	N = 219 (%)	(*p* value)
**Falls**						
Walker present/used	43 (16.8)	10 (4.6)	<0.01	34 (13.8)	9 (4.1)	<0.01
Accessible rooftop without railing	54/110 * (49.1)	33/78 * (42.3)	0.36	42/77 * (54.6)	35/66 * (53.0)	0.86
**Drowning**						
Open buckets of water	134 (52.3)	63 (29.2)	<0.01	106 (42.9)	43/217 * (19.8)	<0.01
Uncovered large vat of water	74 (28.9)	23 (10.7)	<0.01	35 (14.2)	17 (7.8)	0.03
**Burns**						
Water heater within reach	72 (28.1)	9 (4.2)	<0.01	65 (26.3)	4/218 * (1.8)	<0.01
Stove within reach	165 (64.5)	82 (38.1)	<0.01	114 (46.2)	47 (21.5)	<0.01
Matches within reach	127 (49.6)	68 (31.5)	<0.01	94 (38.1)	39 (17.8)	<0.01
Open fire within reach	65 (25.4)	37 (17.1)	0.03	19 (7.7)	30 (13.7)	0.04
Iron within reach	146 (57.0)	87 (40.5)	<0.01	81 (32.8)	92 (42.0)	0.04
Overloaded outlets	47 (18.4)	42 (19.4)	0.76	47 (19.0)	38 (17.4)	0.64
Frayed/loose cords within reach	44 (17.2)	24 (11.1)	0.06	42 (17.0)	18 (8.2)	<0.01
**Poisoning**						
Cooking fluids labeled inappropriately	81 (31.6)	67 (31.0)	0.89	35 (14.2)	38 (17.4)	0.35
Cleaning supplies within reach	86 (33.6)	45 (20.9)	<0.01	84 (34.0)	27 (12.3)	<0.01
Shampoos/soaps within reach	99 (38.7)	28 (13.0)	<0.01	58 (23.5)	26 (11.9)	<0.01
Medicines within reach	47 (18.4)	12 (5.6)	<0.01	30 (12.2)	17 (7.8)	0.12
**Lacerations**						
Breakable objects within reach	88 (34.4)	21 (9.7)	<0.01	65 (26.3)	24/218 * (11.0)	<0.01
Knives within reach	110 (43.0)	47 (21.8)	<0.01	76 (30.8)	22 (10.1)	<0.01
Fan/sharp object within reach	129 (50.4)	89 (41.4)	0.05	113 (45.8)	82 (37.4)	0.07

* Denominators less than the full N denote the total for whom the question was relevant or occasions when a question was missed.

In order to address the intervention results in light of the significant demographic differences between the two groups, we calculated the odds of a household having changed from the presence of a hazard in the pre-intervention assessment to lack of that hazard in the post-intervention assessment, while controlling for various demographic variables including level of maternal education and relationship of primary caregiver. The educational pamphlet group served as the reference group (Table 3). Results demonstrated that the households in the in-home tutorial group had a higher odds of no longer having an open vat of water (OR 2.14, 95% CI: 1.28, 3.58), an open fire within reach of a child (OR 3.55, 95% CI: 1.80, 7.00), an iron within reach of a child (OR 1.95, 95% CI: 1.21, 3.12), inappropriately labeled cooking fluid containers (OR 1.86, 95% CI: 1.07, 3.25), and shampoos/soaps within reach of the child (OR 1.78, 95% CI: 1.12, 2.82). Alternately, the educational pamphlet group had trend of improvement of having overloaded outlets (OR 0.75, 95% CI: 0.43, 1.30), frayed/loose cords within reach of a child (OR 0.84, 95% CI: 0.48, 1.47), and cleaning supplies within reach of a child (OR 0.86, 95% CI: 0.54, 1.37); however none of these were statistically significant.

**Table 3:** Odds of change from presence to lack of specific hazards in the in-home tutorial group as opposed to the educational pamphlet group.

Hazard Description	Odds Ratio (OR) *	95% CI
**Falls**		
Walker present/used	1.48	0.84, 2.62
Accessible rooftop without railing	1.04	0.61, 1.79
**Drowning**		
Open bucket of water	1.27	0.83, 1.95
Uncovered vat/pool of water	2.14	1.28, 3.58
**Burns**		
Water heater within reach	1.20	0.76, 1.88
Stove within reach	1.09	0.72, 1.67
Matches within reach	1.07	0.69, 1.66
Open fire within reach	3.55	1.80, 7.00
Iron within reach	1.95	1.21, 3.12
Overloaded outlets	0.75	0.43, 1.30
Frayed/loose cords within reach	0.84	0.48, 1.47
**Poisoning**		
Cooking fluids labeled inappropriately	1.86	1.07, 3.25
Cleaning supplies within reach	0.86	0.54, 1.37
Medicines within reach	1.45	0.82, 2.55
Shampoos/soaps within reach	1.78	1.12, 2.82
**Cuts**		
Breakable objects within reach	1.52	0.96, 2.41
Knives within reach	1.11	0.71, 1.72
Fan/sharp objects within reach	1.17	0.73, 1.89

* Variables being controlled for in the Multivariate Logistical Regression model: level of maternal education, relationship of primary caregiver, child’s gender, child’s age, and respondent age.

## 4. Discussion

Our study showed an overall significant decrease in 13 out of 18 potential hazards for all major types of unintentional injuries following the conduct of a home hazard assessment and provision of prevention information, either in the form of an educational pamphlet that the caregiver(s) could read on their own or an in-home tutorial done jointly with a caregiver and a study data collector. Whether the changes were due to new information gained by the caregivers or simply the pointing out of safety hazards during the conduct of the hazard assessment is unclear; either way, in the immediate 4–5 months following this intervention, there were fewer childhood hazards present in these low-income housing communities in Karachi, Pakistan. Our findings are similar to what other studies and meta-analyses have found following home visits in other settings [10,12,13,14,15] in that home visits and information dissemination can result in behavior change regarding presence of injury hazards in the home.

The analysis of the odds of a positive change in hazard presence (that is, going from presence of a hazard in the initial assessment to lack of that hazard present in the follow up assessment) allowed a direct comparison of the two interventions while controlling for the various demographic variables including those that differed significantly between the two groups. This analysis showed that the odds of change were statistically significantly higher for many hazards in the in-home tutorial group *versus* the educational pamphlet group. In contrast, the hazards which had a higher odds of change in the educational pamphlet group did not reach statistical significance. Therefore, our study suggests that the use of an in-home tutorial may be a more effective method for the dissemination of childhood hazard prevention methods. This could be because of the added benefit of having someone point out hazards and talk through possible reduction strategies with caregivers as opposed to simply handing a caregiver a pamphlet. However, further comparison studies will be needed in order to establish the optimum strategies for injury prevention information dissemination in LMIC settings.

Although we employed general data collectors for the implementation of the intervention, health workers (HWs) already involved in delivering health information to communities could easily be trained to implement these interventions. Given the widespread use of health workers in several health-related areas (including infectious and chronic diseases) in many countries [25,26,27,28], this study suggests a tremendous potential for the impact of using trained HWs for the dissemination of injury prevention information in LMIC settings. Incorporating injury prevention into the topic areas covered by community health workers has been suggested in a few contexts previously [29,30].

Our study had several limitations. First, enrollees were not randomized to an intervention, which likely contributed to the significant differences between the two neighborhoods. Ideally, a follow up trial should be considerably larger in which several communities or neighborhoods could be cluster-randomized to receiving each of these interventions. Second, the two groups differed significantly in level of respondent education and the relationship of the primary caregiver to the child. Therefore, it might have been predicted that the educational pamphlet group had a lower prevalence of most home hazards prior to any intervention. However, it is important to note that hazard improvement after *versus* before the intervention occurred in both groups. Third, we assessed only changes in presence of hazards, but did not demonstrate a difference in incidence of unintentional injuries. We are in the process of conducting a longer-term follow up (15–18 months following the initial intervention) in which we will assess both ongoing presence of hazards as well as recent unintentional injury history in the index children. Fourth, we utilized the same individuals (DCs) to do the hazard assessments as well as implement the interventions. While this facilitated building up a necessary rapport with the families, it did introduce a potential bias in the hazard assessments if the DCs had pre-conceived notions about the benefits of one intervention strategy *versus* the other. Future studies should consider utilizing different people for hazard assessment conductors *versus* interventionalists. Finally, there was a 10–15% loss to follow up between the initial and follow-up assessments. While this was accounted for in our sample size calculations, every effort should be made to minimize this gap in future studies.

## 5. Conclusions

Our study supports the findings from many studies from HICs and a few studies in LMICs that have demonstrated a significant change in knowledge and presence of injury hazards following dissemination of targeted childhood injury prevention information [10,12,13,14,15]. In this pilot study, it appeared that in-home tutorials are the most effective method for information dissemination, although the use of an educational pamphlet also showed positive impact. There are of course other considerations (resource needs, literacy requirements, *etc.*) that should be taken into account when choosing to use an in-home tutorial or an educational pamphlet in other areas within Pakistan or in other LMIC settings. Future studies in LMIC settings should focus on assessment of both of these types of tools, and also explore the use of educational pamphlets by healthcare providers as is commonly done in HICs. A larger randomized trial could be helpful in comparing these two information dissemination methods; future trials should address the limitations encountered in this study as well as conduct pre-trial assessments of the target populations to assess characteristics such as literacy level.

The development of effective home hazard reduction educational materials could have significant impact on the burden of home injuries in children particularly in lower-income countries such as Pakistan. These advances would heed the call by the World Health Organization for more targeted interventions to address the global burden of child injuries.

## References

[1] Mock C., Peden M., Hyder A.A., Butchart A., Krug E. (2008). Child injuries and violence: The new challenge for child health. Bull. WHO.

[2] Peden M. (2008). World report on child injury prevention appeals to “keep kids safe”. Inj. Prev..

[3] Zia N., Khan U.R., Razzak J.A., Puvanachandra P., Hyder A.A. (2012). Understanding unintentional childhood home injuries: Pilot surveillance data from Karachi, Pakistan. BMC Res. Notes.

[4] Sengoelge M., Hasselberg M., Laflamme L. (2011). Child home injury mortality in Europe: A 16-country analysis. Eur. J. Public Health.

[5] Mahalakshmy T., Dongre A.R., Kalaiselvan G. (2011). Epidemiology of childhood injuries in rural Puducherry, South India. Indian J. Pediatr..

[6] Irving L. (2011). Preventing unintentional injuries in children and young people under 15. Community Pract..

[7] Gururaj G. (2012). Injury prevention and care: An important public health agenda for health, survival and safety of children. Indian J. Pediatr..

[8] Phelan K.J., Khoury J., Xu Y., Liddy S., Hornung R., Lanphear B.P. (2011). A randomized controlled trial of home injury hazard reduction: The home injury study. Arch. Pediatr. Adolesc. Med..

[9] Kendrick D., Coupland C., Mulvaney C., Simpson J., Smith S.J., Sutton A., Watson M., Woods A. (2007). Home safety education and provision of safety equipment for injury prevention. Cochrane Database Syst. Rev..

[10] Kendrick D., Elkan R., Hewitt M., Dewey M., Blair M., Robinson J., Williams D., Brummell K. (2000). Does home visiting improve parenting and the quality of the home environment? A systematic review and meta analysis. Arch. Dis. Child..

[11] Elkan R., Kendrick D., Hewitt M., Robinson J.J., Tolley K., Blair M., Dewey M., Williams D., Brummell K. (2000). The effectiveness of domiciliary health visiting: A systematic review of international studies and a selective review of the British literature. Health Technol. Assess..

[12] Babul S., Olsen L., Janssen P., McIntee P., Raina P. (2007). A randomized trial to assess the effectiveness of an infant home safety programme. Int. J. Inj. Contr. Saf. Promot..

[13] Odendaal W., van Niekerk A., Jordaan E., Seedat M. (2009). The impact of a home visitation programme on household hazards associated with unintentional childhood injuries: A randomised controlled trial. Accid. Anal. Prev..

[14] Carlsson A., Bramhagen A.C., Jansson A., Dykes A.K. (2011). Precautions taken by mothers to prevent burn and scald injuries to young children at home: An intervention study. Scand. J. Public Health.

[15] Swart L., van Niekerk A., Seedat M., Jordaan E. (2008). Paraprofessional home visitation program to prevent childhood unintentional injuries in low-income communities: A cluster randomized controlled trial. Inj. Prev..

[16] Nansel T.R., Weaver N.L., Jacobsen H.A., Glasheen C., Kreuter M.W. (2008). Preventing unintentional pediatric injuries: A tailored intervention for parents and providers. Health Educ. Res..

[17] Sznajder M., Leduc S., Janvrin M.P., Bonnin M.H., Aegerter P., Baudier F., Chevallier B. (2003). Home delivery of an injury prevention kit for children in four french cities: A controlled randomized trial. Inj. Prev..

[18] Pakistan: Country at a Glance. www.worldbank.org/en/country/pakistan.

[19] Lasi S., Rafique G., Peermohamed H. (2010). Childhood injuries in Pakistan: Results from two communities. J. Health Popul. Nutr..

[20] Fatmi Z., Kazi A., Hadden W.C., Bhutta Z.A., Razzak J.A., Pappas G. (2009). Incidence and pattern of unintentional injuries and resulting disability among children under 5 years of age: Results of the national health survey of Pakistan. Paediatr. Perinat. Epidemiol..

[21] Hyder A.A., Wali S., Fishman S., Schenk E. (2008). The burden of unintentional injuries among the under-five population in South Asia. Acta Paediatr..

[22] Hyder A.A., Sugerman D.E., Puvanachandra P., Razzak J., El-Sayed H., Isaza A., Rahman F., Peden M. (2009). Global childhood unintentional injury surveillance in four cities in developing countries: A pilot study. Bull. WHO.

[23] Rehmani R., Leblanc J.C. (2010). Home visits reduce the number of hazards for childhood home injuries in Karachi, Pakistan: A randomized controlled trial. Int. J. Emerg. Med..

[24] Hyder A.A., Chandran A., Khan U.R., Zia N., Huang C.M., de Ramirez S.S., Razzak J. (2012). Childhood unintentional injuries: Need for a community-based home injury risk assessments in Pakistan. Int. J. Pediatr..

[25] Wong C.X., Carson K.V., Smith B.J. (2012). Home care by outreach nursing for chronic obstructive pulmonary disease. Cochrane Database Syst. Rev..

[26] Viswanathan M., Kraschnewski J., Nishikawa B., Morgan L.C., Thieda P., Honeycutt A., Lohr K.N., Jonas D. (2009). Outcomes of Community Health Worker Interventions.

[27] Viswanathan M., Kraschnewski J.L., Nishikawa B., Morgan L.C., Honeycutt A.A., Thieda P., Lohr K.N., Jonas D.E. (2010). Outcomes and costs of community health worker interventions: A systematic review. Med. Care.

[28] Lewin S., Munabi-Babigumira S., Glenton C., Daniels K., Bosch-Capblanch X., van Wyk B.E., Odgaard-Jensen J., Johansen M., Aja G.N., Zwarenstein M. (2010). Lay health workers in primary and community health care for maternal and child health and the management of infectious diseases. Cochrane Database Syst. Rev..

[29] Forst L., Lacey S., Chen H.Y., Jimenez R., Bauer S., Skinner S., Alvarado R., Nickels L., Zanoni J., Petrea R. (2004). Effectiveness of community health workers for promoting use of safety eyewear by Latino farm workers. Am. J. Ind. Med..

[30] Marsh P., Kendrick D., Williams E.I. (1995). Health visitors’ knowledge, attitudes and practices in childhood accident prevention. J. Public Health Med..

